# Molecular Responses to Cadmium Exposure in Two Contrasting Durum Wheat Genotypes

**DOI:** 10.3390/ijms22147343

**Published:** 2021-07-08

**Authors:** Erika Sabella, Andrea Luvisi, Alessandra Genga, Luigi De Bellis, Alessio Aprile

**Affiliations:** Department of Biological and Environmental Sciences and Technologies, University of Salento, via Prov.le Monteroni 165, 73100 Lecce, Italy; erika.sabella@unisalento.it (E.S.); andrea.luvisi@unisalento.it (A.L.); alessandra.genga@unisalento.it (A.G.); luigi.debellis@unisalento.it (L.D.B.)

**Keywords:** *Triticum durum*, heavy metals, mRNA sequencing, nicotianamine synthase, mugineic acid, basic helix–loop–helix (bHLH)

## Abstract

Cadmium is a heavy metal that can be easily accumulated in durum wheat kernels and enter the human food chain. Two near-isogenic lines (NILs) with contrasting cadmium accumulation in grains, High-Cd or Low-Cd (H-Cd NIL and L-Cd NIL, respectively), were used to understand the Cd accumulation and transport mechanisms in durum wheat roots. Plants were cultivated in hydroponic solution, and cadmium concentrations in roots, shoots and grains were quantified. To evaluate the molecular mechanism activated in the two NILs, the transcriptomes of roots were analyzed. The observed response is complex and involves many genes and molecular mechanisms. We found that the gene sequences of two basic helix–loop–helix (bHLH) transcription factors (bHLH29 and bHLH38) differ between the two genotypes. In addition, the transporter Heavy Metal Tolerance 1 (HMT-1) is expressed only in the low-Cd genotype and many peroxidase genes are up-regulated only in the L-Cd NIL, suggesting ROS scavenging and root lignification as active responses to cadmium presence. Finally, we hypothesize that some aquaporins could enhance the Cd translocation from roots to shoots. The response to cadmium in durum wheat is therefore extremely complex and involves transcription factors, chelators, heavy metal transporters, peroxidases and aquaporins. All these new findings could help to elucidate the cadmium tolerance in wheat and address future breeding programs.

## 1. Introduction

Heavy metals naturally occur in the soil as rare elements; however, their amounts are increasing due to human activities which continuously aggravate environmental pollution. Among the heavy metals, cadmium (Cd) is one of the most toxic, both for human health and for plants [1,2]. For humans, usually, the primary source of exposure is the dietary ingestion of contaminated crops [3]. Plants promptly take up Cd from soil since it has a high solubility in water and, even at low concentrations, it can cause phytotoxic effects (i.e., reduction of seed germination, early seedling growth and plant biomass. Other examples of phytotoxic effects are the decrease in photosynthesis rate, transpiration rate, stomatal conductance, etc.) [4,5]. The mechanisms of Cd biotoxicity are the result of multiple biological effects on membranes, on mitochondrial structure and function, on DNA and on gene expression. Although most of the biological effects of cadmium are linked to its ability to modulate the redox state of the cell, certain effects may relate to the structural similarities between Cd and calcium [6]. On the other hand, plants have evolved several mechanisms for Cd detoxification; some previous studies have shown that there are three main strategies that plants can use to overcome Cd toxicity: the first strategy is Cd absorption or isolation inside the plant (plants store the Cd in specific cellular locations inside to reduce the Cd concentration in tissues that are metabolically active); the second one is the alleviation of Cd toxicity by its removal through a series of chelating mechanisms. The third is the removal of the oxygen species (ROS) that accumulate during Cd-induced stress [7]. These plant tolerance mechanisms require a complex coordination of physiological and biochemical processes, including changes in global gene expression.

Transcriptome sequencing is a powerful tool for identifying gene networks from the genome-wide expression analysis of many living species, for example, in response to the stress heavy metals induce. In recent years, an increasing number of transcriptomic studies have been conducted on gene expression changes induced in plants under Cd stress. Chen et al. [8] conducted transcriptome sequencing of the roots of cotton grown under three different Cd concentrations and found that the genes responsive to Cd stress were mainly related to oxidation resistance (upregulation of genes coding for superoxide dismutase, thioredoxin peroxidase and ascorbate peroxidase, all involved in ROS removal), detoxification complexation (genes coding for glutathione transferase and metallothionein were upregulated) and differentially expressed transcription factors (20 were upregulated, including MYB, zinc finger, leucine zipper and NAC transcription factors). In bread wheat (*T. aestivum* L.) and in *T. Urartu*, RNA sequencing experiments have revealed the upregulation of many genes in roots, especially genes involved in phenylpropanoid and lignin pathways [9,10].

Induction of genes involved in sulfur assimilation and glutathione (GSH) metabolism was also observed in Arabidopsis roots exposed to Cd; in leaves, an early induction of several genes encoding enzymes of phenylpropanoid biosynthesis was observed. Even in root of Arabidopsis exposed to Cd, the gene *At5g13080*, encoding a WRKY family transcription factor, was found to be strongly induced [11]. The comparison of the data of transcriptome profiles from several plant species exposed to Cd treatment showed that phenylpropanoid biosynthesis and glutathione metabolism are the main pathways in response to Cd stress [12]; furthermore, several transcription factors were strongly induced by Cd stress [13,14,15]. For example, in the bark of *Populus x canescens*, several transcription factors, such as WRKY70/53, MYB94, ABI5 and AP2, were activated in response to Cd stress [13]. 

Among the important agricultural crops for the human diet, durum wheat (*Triticum turgidum* L. ssp. *durum* (Desf.) Husn) accumulates high Cd concentrations in the grains, and they often exceed international trade standards [16]. The different Cd accumulation levels in durum wheat cultivars are well known [16,17,18] but the genetic factors and the molecular mechanisms conferring the low-Cd phenotype are still unknown. Phenotyping the trait “low-Cd ingrain” is time consuming and costly, so genomic tools/markers associated with Cd could be a more efficient approach for developing cultivars with low Cd levels and to reduce the Cd level in durum wheat-based products (e.g., semolina, pasta). In durum wheat, a single major locus for Cd concentration in grain has been reported on chromosome 5BL [19] and it has been designated as *Cdu-B1*. The molecular marker ScOPC20 [20] is linked to *Cdu-B1* [19] and it can be used to screen large wheat populations. Wiebe et al. [21] have mapped the *Cdu-B1* locus and, subsequently, [22] they saturated the locus with molecular markers, leading to the identification of the candidate Cd uptake gene *TdHMA3-B1*. 

Physiologically, Cd enters the plant from the soil through the roots and it is then translocated to the upper tissues of the plants by the xylem and, presumably via the phloem, it is then mobilized to the developing grains. According to Maccaferri et al. [23], the gene *TdHMA3-B1* may be associated with a process that influences Cd sequestering in the roots. However, AbuHammad et al. [24] showed the presence of a major QTL contributing low Cd uptake on chromosome 2B and Oladzad-Abbasabadi et al. [25] reported the novel low Cd uptake gene *Cdu2-B* in the durum experimental line D041735. In addition, several studies have shown that a specific trait can be regulated by different genetic factors among different genotypes [26]. 

RNA sequencing studies connect genomic data and biological function, which facilitates finding genes involved in regulation associated to stress adaptation, detoxification, growth and development. In this study, root transcriptomic analysis of Cd-treated or untreated low-Cd and high-Cd near-isogenic lines of durum wheat was carried out to reveal genes and metabolic routes involved in Cd uptake, translocation, and detoxification. Our data could be helpful to uncover the molecular mechanisms related to low Cd accumulation in durum wheat.

## 2. Results and Discussion

### 2.1. Biomass and Cd Concentration in Plant Tissues

**Figure 1:**
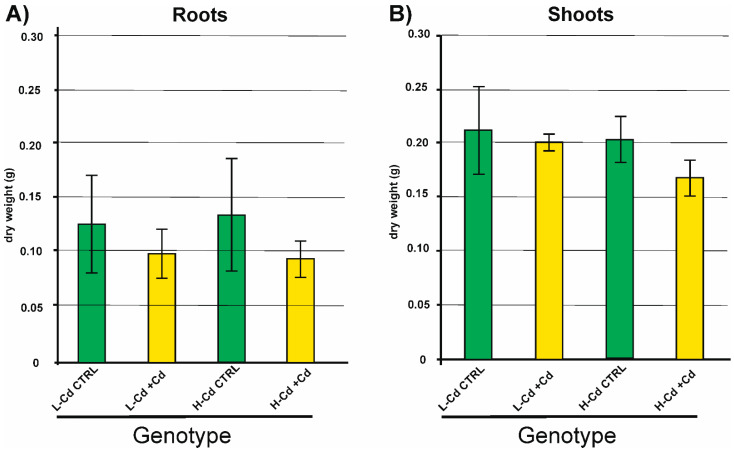
Biomass of root (**A**) and shoot (**B**) tissues of H-Cd and L-Cd NILs cultivated in hydroponic solution with Cd 0.5 µM (+Cd) or without Cd (CTRL). Tissues were sampled 50 days after germination. Statistical analysis (two-way ANOVA) did not show any relevant difference (*p*-value < 0.05, *n* = 3).

The Cd concentration of 0.5 μM in the hydroponic solution was not toxic for the durum wheat plants as reported previously by other authors [27]. In fact, the growth and biomass production were not significantly different between control and cadmium-treated plants as demonstrated by the analysis of variance (two-way ANOVA, *p*-value < 0.05, *n* = 3) (Figure 1).

**Figure 2:**
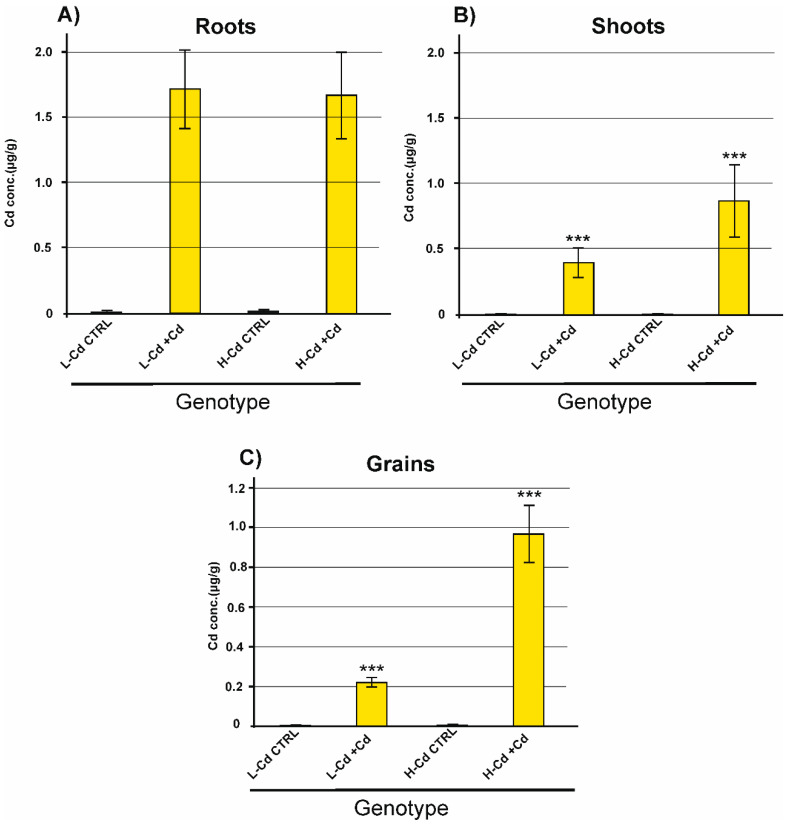
Cd concentrations in durum wheat tissues of H-Cd and L-Cd NILs cultivated in hydroponic solution with Cd 0.5 µM (+Cd) or without Cd (CTRL). Root and shoot tissues were sampled 50 days after germination (panels (**A**,**B**)). Grains were sampled at maturity (panel (**C**)). ANOVA statistical analysis (*** = *p*-value < 0.001, *n* = 3).

As expected, the Cd concentration was higher in roots than in shoots (Figure 2). Additionally, the Cd concentration in the roots of the two wheat genotypes was similar, whereas the concentration in shoot tissues was higher for the H-Cd NIL (about two times higher, Figure 2). L-Cd and H-Cd NILs are very similar plants (root and leaf size) and they have similar transpiration rates [27] and, therefore, the different Cd accumulation levels cannot be due to differences in water fluxes in the plants. These data suggest a more efficient Cd translocation or a less efficient retention in roots, regulated by specific proteins or metabolic pathways.

At the end of the plant cycle, as for shoot tissues, the kernels of the H-Cd NIL showed a high Cd concentration: 0.97 ± 0.14 µg/g, about five times higher in comparison to L-Cd NIL grains (Figure 2). 

### 2.2. RNA Sequencing and Quality of Data

The Illumina HiSeq2500 device produced 721.9 million (M) single-end reads with a median of 21.9 M reads/sample (min 19.0 M, max 29.1 M). For each contig, the relative abundance was calculated as fragments per kilobase million (FPKM) (Table S1: “Expression data”). To evaluate the quality of the sequencing data, the Pearson correlation coefficients among biological replicates were calculated. The values ranged from 0.943 to 0.995, representing a high level of correlation among biological replicates, as expected. 

**Figure 3:**
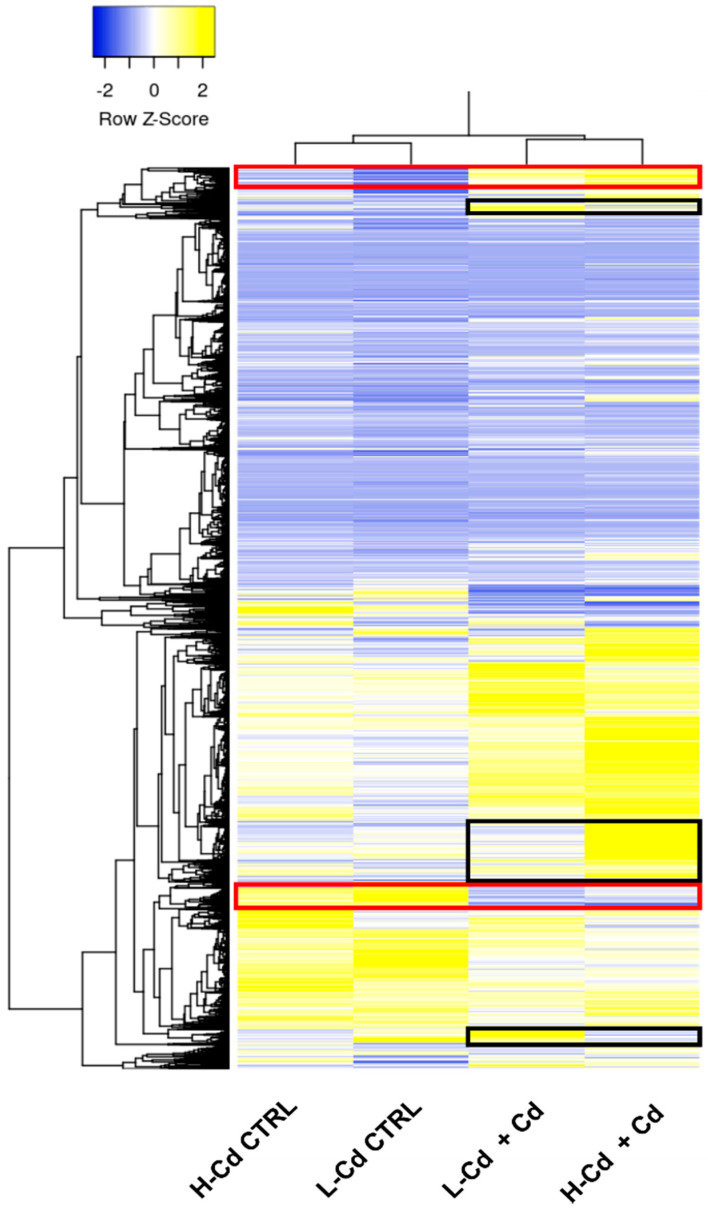
Heatmap of the unsupervised clustering of the transcriptome data of H-Cd and L-Cd NILs after treatment with Cd in hydroponic solution. The two main branches separate CTRL samples from Cd-treated samples, suggesting that the treatment is the main source of variation in the data set.

Figure 3 shows an unsupervised clustering in the form of a heatmap. The Cd treatment is the main source of variation in the data set since CTRL samples and treated samples clustered separately. In the heatmap, several areas, highlighted with red or black boxes, indicate gene clusters whose expression is treatment specific (red boxes) or genotype- specific (black boxes). The upper red box groups genes whose expression is higher in Cd-treated samples, whereas the lower red box highlights genes downregulated by cadmium treatment. The three black boxes show gene clusters upregulated only in one of the two genotypes. These preliminary data suggest a complex response to cadmium treatment and a significant transcriptome modulation. Previously, genetic approaches such as QTL analysis have reported the presence of a single locus as the basis of cadmium accumulation in wheat [19,22,25]. On the contrary, our data suggest a significant transcriptomic re-organization after Cd treatment that cannot be explained with the simple regulation of a single locus or gene.

### 2.3. Hypothesis-Based Analysis of the Transcriptome Modulation in NILs Treated with Cadmium

The analysis of the transcriptome of the two NILs grown in the absence or presence of Cd showed that the roots are able to express about 30 k contigs. In the CTRL condition, L-Cd NIL and H-Cd NIL roots activated the transcription of about 33 k contigs, whereas this number decreased after the treatment with Cd to about 29.5 k contigs (Table S1: “Expression data”). 

These numbers suggest great complexity and the regulation/activation of homologous genes in response to cadmium treatment.

The experiments based on the transcriptome analysis usually follow a holistic approach and tend to show the variations of expression of large gene categories that move together (gene clustering), count the number of transcripts which are equivalently regulated in multiple samples (Venn diagrams) or find over-represented functional categories among specific groups of transcripts.

Sometimes, the outputs of these approaches are very complex, and the biological meaning of such a huge data set may be not clear. For this reason, to unravel the complex molecular responses activated or downregulated by cadmium treatment, we formulated several hypotheses to reveal genes involved in the uptake and/or transport of the heavy metal in roots. 

The near-isogenic lines H-Cd (TL 8982-H) and L-Cd (TL 8982-L) [28] derive from the crossing and backcrossing between Kyle and Nile cultivars: Nile is a landrace collected by the International Center of Agricultural Research in the Dry Areas, Syria, showing low Cd accumulation in grains [25], whereas Kyle is a highly Cd accumulating cultivar. The genetic similarity of the two NILs (more the 95% of their genomes) and the marked phenotypic difference in relation to cadmium accumulation allowed us to carry out useful comparisons with the aim to identify those elements causing the different cadmium accumulation levels in the two genotypes.

**Hypothesis** **1 (H1).** *Genes Constitutively Expressed in the L-Cd Genotype, But Not Expressed in the H-Cd NIL.*

The search for genes constitutively expressed in the L-Cd NIL and not expressed at all in the H-Cd NIL has the purpose of identifying possible genetic elements that explain the different behavior between the L-Cd NIL and the H-Cd NIL. If a gene is expressed in the L-Cd NIL, but not in H-Cd NIL, it could be involved in avoiding Cd translocation to the upper organs.

By applying filters on the expression level (L-Cd > 25 FPKM and H-Cd < 5 FPKM), five genes were identified (Table 1). Two of them have no annotation, and the others are annotated as a cyclophilin, a sulfurtransferase and an ABC transporter protein (also named heavy metal tolerance factor 1—HMT1). The last gene (*contig9490*), coding for a transporter, could be involved in cadmium accumulation in the roots. As observed by Ortiz et al. [29] in *Schizosaccharomyces pombe*, HMT1 is an ATP-dependent transporter of Cd–phytochelatin complexes into the vacuole. The presence of a similar HMT1 in different species, such as *Schizosaccharomyces pombe, Chlamydomonas reinhardtii*, *Caenorhabditis elegans*, *Drosophila melanogaster*, *Rattus norvegicus* and *Homo sapiens*, suggests the conservation of this gene among species [30].

The ectopic expression of *HMT1* in *Arabidopsis thaliana* enhances the tolerance to Cd, copper, arsenic and zinc and their accumulations in roots. Moreover, the root-specific expression of *SpHMT1* reduced the Cd and copper levels in seed by half, and arsenic to one-third, in comparison to the wild type. The molecular mechanism proposed was the accumulation of heavy metals in root vacuoles [31]. Therefore, *contig9490*, which is expressed in L-Cd NIL but not in the H-Cd line, represents a good candidate to explain the differences between the two genotypes.

**Hypothesis** **2 (H2).** *Genes Constitutively Expressed in the H-Cd Genotype, But Not Expressed in the L-Cd NIL.*

The second hypothesis concerns genes whose expression was high in H-Cd NIL and were not expressed in the L-Cd NIL (H-Cd > 25 FPKM and L-Cd < 5 FPKM). Genes with these expression properties could explain the better capacity of the H-Cd NIL to translocate Cd from roots to shoots. Nine contigs were identified (Table 2).

The more interesting genes are *contig13191*, an aquaporin pip1-2-like (*AT1G01620*), and *contig17660*, which corresponds to aquaporin pip1-5 (*AT4G00430*). The H-Cd NIL plants negatively modulate the expression of these genes following Cd treatment, while the two genes are practically not expressed in the L-Cd NIL regardless of the presence of cadmium. Consequently, once cadmium stress has been perceived, only H-Cd roots activate a downregulation of two aquaporins, probably to reduce the uptake of Cd together with water. On the contrary, the absence of constitutive expression in the L-Cd line could be a benefit, limiting the intake of water containing the heavy metal. Additionally, a massive downregulation of aquaporins in a low cadmium-accumulating *Solanum torvum* was observed by Yamaguchi et al. [32].

The downregulation of *contig13191* and *contig17660* in the H-Cd NIL and the lack of expression in the L-Cd NIL suggested that aquaporins and water balance adjustment could be efficient mechanisms in durum wheat cadmium tolerance.

**Hypothesis** **3 (H3).** *Genes More Expressed in the CTRL Samples of L-Cd Than H-Cd NILs.*

The third hypothesis concerns the possibility that genes more highly expressed in the L-Cd genotype than in the H-Cd NIL are responsible for the different behavior of the two NILs in relation to Cd. This hypothesis is close to hypothesis 1, but in this case the genes are expressed in both wheat lines. To individuate these genes, we applied two filters. The first filter selected genes with a strong expression (five times the background expression level) in L-Cd NILs to remove weakly induced genes; the second filter was based on the statistical difference between the expression of CTRL samples of L-Cd and H-Cd NILs. The results are shown in Table S2, “More expressed in L-Cd CTRL”, that includes a list of 292 contigs. The list was checked both manually and by searching for over-represented categories (*p*-value < 0.05) using g:Profiler software [33].

We did not find any over-represented category, and after the manual check of the gene list, only one interesting contig (*contig68204*) was found to be putatively involved in cell transport, detoxification or other mechanisms involved in cadmium response, because its annotation was aquaporin nip2-1-like (*AT4G18910*) and the expression level was less than 2.5-fold between H-Cd and L-Cd NILs. Due to the slight difference in expression between the two wheat lines, we do not imagine a significant role for this contig in Cd accumulation/translocation.

**Hypothesis** **4 (H4).** *Genes More Expressed in the CTRL Samples of H-Cd Than L-Cd NILs.*

Hypothesis 4: the genes that are more expressed in CTLR samples of the H-Cd NIL than the L-Cd NIL could be responsible for the differences between the two NILs. Applying the same but inverse condition of hypothesis 3, 1328 contigs were identified (Table S3: “More expressed in H-Cd CTRL”). 

This gene list was long, but the bioinformatic analysis did not reveal any over-represented specific gene category somehow linked to Cd uptake/translocation. Scrolling through the list starting from the highest fold change (Table S3: “More expressed in H-Cd CTRL”), we found genes annotated as cell wall kinase, adenine hydrolase, wounding-related protein and early nodulin-like protein. These gene annotations seem unrelated to cadmium response. These genes probably simply fall in an unshared region of the NIL genomes and, consequently, they have different expression regulation.

The first interesting contigs in the “hypothesis 4” gene list were *contig6432* and *contig13016* (both annotated as nicotianamine synthase 4). The fold change between CTRL samples was about 15. These contigs were also characterized by a strong upregulation after cadmium treatment: in the H-Cd NIL, the upregulation was about 3-fold, but in the L-Cd NIL, it was almost 200-fold (Table S3: “More expressed in H-Cd CTRL”), suggesting a role for these contigs in the cadmium response. These genes will be further described in Hypothesis 7.

The “hypothesis 4” comparison allowed us to understand that, although the two near-isogenic lines can be defined as genetically similar, the transcriptomes show many differences, but which do not fall into categories of particular interest and cannot be traced back to the differential accumulation of cadmium. These gene expression differences are probably due to genomic differences between the two NILs.

**Hypothesis** **5 (H5).** *Genes Upregulated and Downregulated Only in the L-Cd NIL by Cadmium Treatment.*

The transcriptome analysis of the isogenic lines revealed that there are 665 contigs that are upregulated by the Cd treatment only in the L-Cd NIL (Table S4: “Upregulated only in L-Cd treated plants”), a large group of genes that could be the basis of the different behavior between the L-Cd and H-Cd NILs.

A functional classification was done using the g:Profiler bioinformatic tool [33]. Two gene categories were found to be over-represented. The first one is called “transmembrane transporter activity”, and 71 out of 665 contigs fall into this category (*p*-value < 0.05). The activation of membrane transporters was quite significant and different kinds of transporters were included. Some of them were linked to cell trafficking (aquaporin, phosphate transporters, sugar transporters, etc.), but many others were related to metal ion transport. Ten contigs were annotated as membrane ATPase transporters (such as HMA5, Heavy Metal Transporter 5), three contigs as ZIP metal ion transporters, four contigs as Yellow Stripe Like (YSL—a protein involved in the transport of the nicotianamine phytosiderophores) and two as vacuolar iron transporters. This huge activation of different transporters indicates that one of the main strategies employed by the L-Cd NILs to reduce cadmium levels is the activation of different kind of transporters. 

The second over-represented category, with 96 contigs, was “oxidoreductase activity”, with most of them (55 contigs) annotated as peroxidases, suggesting a strong response to reduce the toxic effect of reactive oxygen species (ROS). However, peroxidases are also involved in the polymerization of lignin precursors [34] and their massive production could be a defence strategy to halt the cadmium apoplastic route. Cell wall lignification was already proposed as a defence strategy, with a central role for H_2_O_2_ as the substrate and signaling molecule [35]. We found the activation of many peroxidases, suggesting that ROS are strongly produced during cadmium stress and they could have a central role in cell wall lignification.

Therefore, the L-Cd NILs under cadmium stress could activate a wide range of different membrane transporters, probably to counteract the effects of the toxic element, to compartmentalize it and to avoid its translocation throughout the root xylem vessels and, at the same time, they are able to activate many peroxidase genes. The H-Cd NIL did not activate these genes, resulting in a higher Cd content in shoot and kernel tissues.

As well as the upregulated genes, the L-Cd NIL could activate cadmium response strategies by gene downregulation. The comparison of the transcriptome highlighted 43 contigs downregulated by Cd only in the L-Cd NIL (Table S5: “Downregulated only in L-Cd treated plants”). The analysis of functional annotations did not report any interesting activity related to Cd response, suggesting that the cadmium response in the L-Cd NIL is probably controlled by upregulation.

**Hypothesis** **6 (H6).** *Genes Upregulated or Downregulated Only in the H-Cd NIL by Cadmium Treatment.*

This comparison reported 403 contigs to be upregulated only in the H-Cd NIL, but any gene was annotated as having a biological role somewhat linked to Cd (Table S6: “Upregulated only in H-Cd treated plants”).

Looking at the downregulated genes only in the H-Cd NIL, we found 692 contigs (Table S7: “Downregulated only in H-Cd-treated plants”). The analysis of functional categories again revealed the category of “oxidoreductase activity” (62 contigs out of 692). In fact, 12 contigs were annotated as peroxidases, while 50 referred to different primary metabolic processes.

**Hypothesis** **7 (H7).** *Genes Commonly Upregulated and Downregulated by Cadmium Treatment in Both Genotypes but to a Different Extent.*

The differences between the behavior of the two NILs could be explained by the genes that are upregulated in both genotypes but at different levels. 

In the H-Cd and L-Cd NILs, 183 contigs commonly upregulated in roots treated with Cd were identified; 41 out of 183 contigs were strongly upregulated only in the L-Cd NIL. 

**Figure 4:**
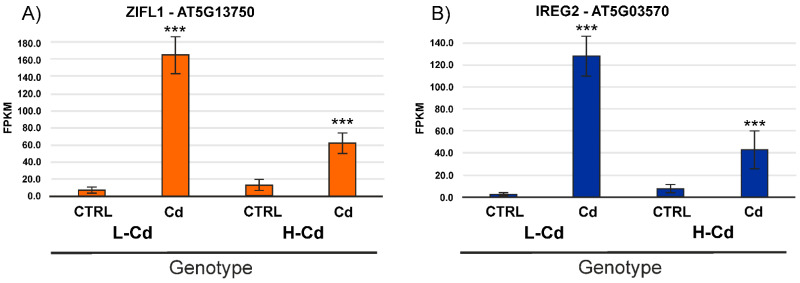
Expression level of *ZIFL1* (Panel **A**) and *IREG2* (Panel **B**). Expression level of the *contig75088* and *contig47439* annotated, respectively, as *ZIFL1* and *IREG2* in roots of the L-Cd and H-Cd NILs grown in standard hydroponic solution (CTRL) and with Cd 0.5 µM (Cd) (*** = *p*-value < 0.001, n = 3).

*Contig75088* and *contig47439* (Table S1: ”Expression data”) are annotated, respectively, as *Zinc Induced Facilitator-Like 1* (*ZIFL1*) (*AT5G13750*) and *Iron Regulated 2* (*IREG2*) (*AT5G03570*), two well-known genes involved in heavy metal translocation in plants [36,37,38]; their level of induction in the L-Cd NIL was very high (Figure 4). *ZIFL1* has high sequence similarity to *Zinc Induced Facilitator* (*ZIF1*) that encodes a major facilitator superfamily (MFS) transporter; it is localized to the tonoplast and like other Zn transporters (Heavy Metal ATP-ase, AtHMA3 and AtHMA4) could transport divalent cations like Zn^2+^ or Cd^2+^ [39,40]. Therefore, a higher expression of ZIFL1 in the L-Cd NIL could enhance Cd translocation into vacuoles or other cellular organelles.

In Arabidopsis, *IREG2* is associated with Cd and nickel tolerance [41] and it is regulated by the bHLH104 transcription factor [37] and by three other bHLH transcription factors: Fer-Like Iron Deficiency-Induced Transcription Factor (FIT), AtbHLH38 and AtbHLH39 [38]. IREG2 is also co-expressed with other heavy metal-related genes such as *HMA3*, *Metal Tolerance Protein* (*MTP*) and *Nicotianamine Synthase* (*NAS*) [37,38], suggesting its key role in heavy metal homeostasis. We observed a 50-fold and five-fold upregulation in the L-Cd and H-Cd NIL, respectively. 

In conclusion, the stronger upregulation of contigs corresponding to *ZIFL1* and *IREG2* in the L-Cd NIL (Figure 4) suggests a role of these two contigs/genes in Cd sequestration at the root level. 

Furthermore, of the 41 contigs that were more upregulated in roots of the L-Cd NIL, 22 are annotated as *nicotianamine synthase* (NAS) genes (Table 3). NAS catalyzes the synthesis of the amino acid nicotianamine (NA) by trimerization of S-adenosyl-methionine (SAM) [42]. 

Plants react to environmental stresses by producing different types of phytochelatins, amino acids and amines (e.g., glycine, histidine, spermidine, putrescine, nicotianamine, mugineic acid) [43]. NA contributes to the uptake of transition metal cations, translocation and homeostasis in cereals. In bread wheat, 21 different NAS genes were identified, and eight of them were located on genome A and five on genome B [44]. In this experiment, the 22 *nicotianamine synthase*-like contigs were annotated as five different NAS genes (*NAS1*, *NAS2*, *NAS3*, *NAS4* and *NAS5B*) and they were strongly upregulated by Cd treatment. The level of expression in the H-Cd NIL was higher than in the L-Cd NIL in CTRL conditions. The opposite situation occurred after the Cd treatment: a higher level of expression in the L-Cd NIL. Additionally, the relative FCs were very different: in the L-Cd NIL, the upregulation was about 160-fold, whereas in the H-Cd NIL, it was about “only” nine-fold.

The upregulation of different contigs related to *NAS* genes, the strong upregulation after Cd treatment and the higher upregulation in the L-Cd NIL suggest that such contigs play a central role in Cd response and that they could also be involved in Cd root compartmentalization.

**Figure 5:**
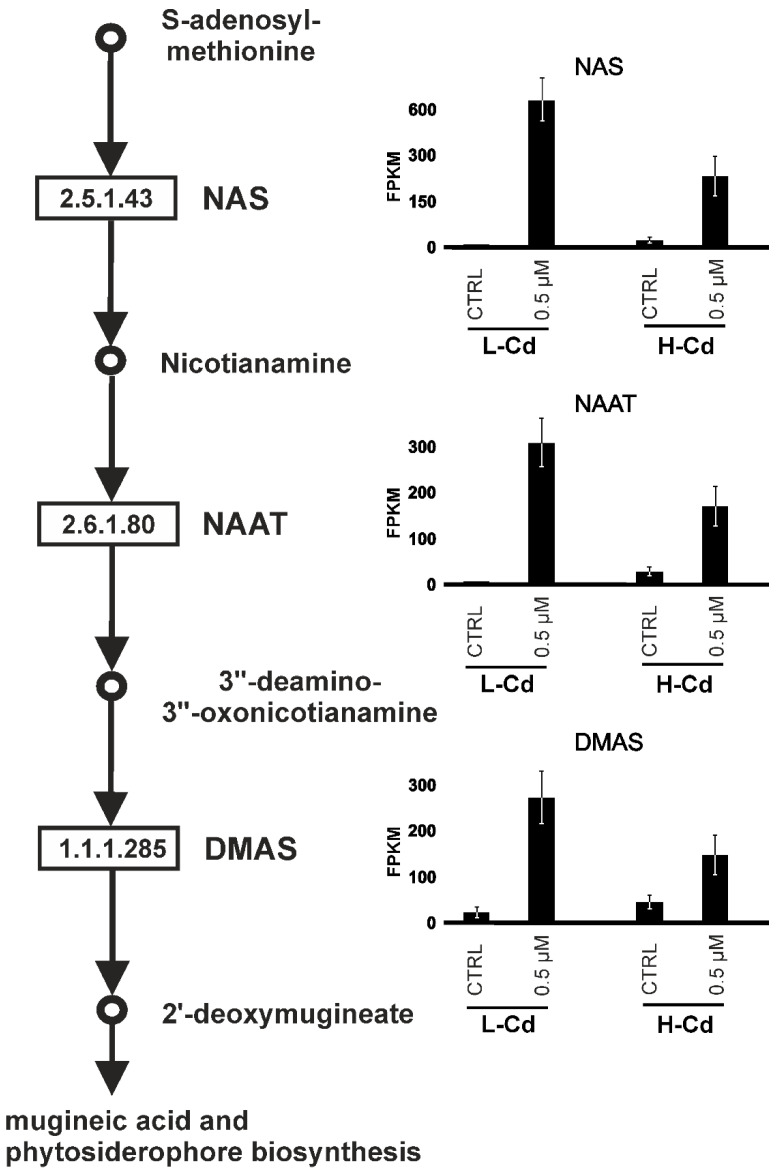
Schematic representation of the mugineic acid pathway. Inferred from MetaCyc [46]. Circles represent the compounds involved in MA pathway. Numbers in the boxes are the KEGG enzyme codes relative to nicotianamine synthase (NAS), nicotianamine amine transferase (NAAT), deoxymugineic acid synthase (DMAS). The genes coding these enzymes are strongly upregulated by cadmium stress in durum wheat and the relative levels of expression are reported on the right.

Nicotianamine is the precursor of metallophores known as mugineic acids (MAs). MA and its derivatives have only been found in graminaceous plants [45]. Mugineic acid is synthesized though nicotianamine deamination followed by reduction to 2′-deoxymugineic acid. Additional hydroxylation reactions generate related phytosiderophores from deoxymugineic acid (Figure 5).

The second and third enzymes of mugineic acid biosynthesis are called nicotianamine aminotransferase (NAAT) and deoxymugineic acid synthase (DMAS). The levels of expression of the genes coding for these two enzymes are reported in Table 4, which shows that these genes are more upregulated in the L-Cd NIL in comparison to the H-Cd NIL: the mean upregulation FC was 6.4 in the H-Cd NIL and about 150 in the L-Cd NIL.

This strong upregulation of the contigs putatively encoding NAAT or DMAS in the low cadmium-translocating genotype suggests a role in cadmium response and could contribute to the explanation of the different behavior between the two NILs. 

Only 71 contigs were commonly downregulated between the L-Cd and H-Cd NILs after the cadmium treatment (Table S1: “Expression data”). The downregulation levels were very similar, so they cannot explain the Cd phenotype differences.

**Hypothesis** **8 (H8).** *Regulatory Genes (Transcription Factors) With Similar Levels of Upregulation But With Different Genome Sequences.*

We found three contigs upregulated both in the L-Cd and H-Cd NILs; they are annotated as bHLH transcription factors (bHLH29, bHLH38 and bHLH47). *Contig49052* (bHLH47) shows an identical nucleotide sequence in the two genotypes, while we found sequence differences in the case of *contig12928* (bHLH29) and *contig59732* (bHLH38).

*Contig12928* has a high sequence similarity to the *Arabidopsis bHLH29* gene (*AT2g28160—Fe-deficiency induced transcription factor—FIT*) and also with the rice *bHLH156-like.* It was upregulated (approximately double) in the cadmium-treated roots of L-Cd and H-Cd NILs (Table S1: “Expression data”). In *Arabidopsis*, it has been observed that the corresponding gene is root specific [47].

*Contig59732* has high sequence similarity to the Arabidopsis transcription factor bHLH38 (OBP3-responsive gene 2—ORG2—*AT3G56970*) and with the rice Iron-Related bHLH Transcription Factor 2 (IRO2). According to previous studies, AtbHLH29/FIT (FER-like Deficiency Induced Transcription Factor) interacts with AtbHLH38/ORG2 to enhance Cd tolerance in *Arabidopsis*, decreasing cadmium transport from roots to shoots and improving the iron homeostasis and regulating the metal concentration in shoots. The same authors also reported that co-overexpression of *FIT* and *ORG2* constitutively activated the expression of *Heavy Metal Associated 3* (*HMA3*) and *Iron Regulated Gene 2* (*IREG2*), which are involved in heavy metal detoxification in *Arabidopsis* [38]. Moreover, co-overexpression of FIT and ORG2 enhanced the expression of *nicotianamine synthase 1* (*NAS1*) and *NAS2*, resulting in the accumulation of nicotiananamine, a crucial chelator for Fe transportation and homeostasis [48]. As reported above, *IREG2* and *NAS* homologue genes were strongly upregulated in our experiment and the upregulation was much stronger in the L-Cd genotype. For this reason, we compared the sequences of this transcription factors to find any sequence differences that could be associated with a different upregulation of *IREG2* and *NAS* genes in the two genotypes. 

**Figure 6:**
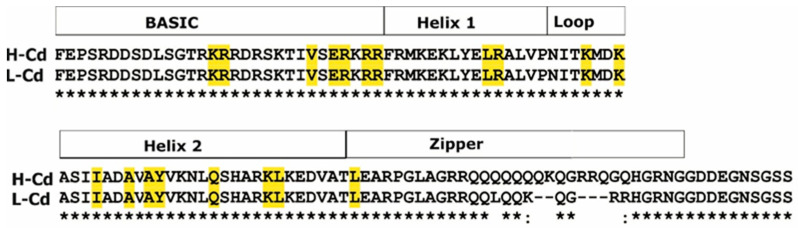
Partial amino acid sequence alignment of H-Cd and L-Cd bHLH29. Conserved residues (identified through model–template alignments by the automated protein structure homology-modeling server SWISS-MODEL) are highlighted in yellow.

The comparison between the *bHLH29* sequence of the H-Cd and L-Cd NILs effectively highlighted a sequence difference: a large deletion of 10 nucleotides in the L-Cd NIL; as consequence of this insertion, amino acid sequences differed in the region of the leucine zipper (ZIP) motif (Figure 6). 

Some bHLH proteins have a leucine zipper (ZIP) motif adjacent to the second helix of the bHLH motif. This domain is predicted to adopt a coiled-coil structure that permits dimerization between proteins. Additionally, in animal bHLH-ZIP proteins, it was demonstrated that the ZIP motif stabilizes protein dimers and that the residues within the ZIP domain determine dimerization specificity [49]. In fact, Wu et al. [38] reported that some bHLH proteins are able to form heterodimers to activate the expression of genes involved in Cd sequestration in Arabidopsis roots. 

**Figure 7:**
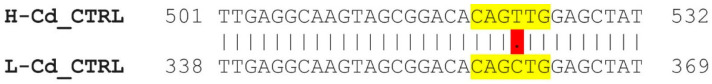
Partial nucleotide sequence alignment of H-Cd and L-Cd *bHLH38*. The E-box consensus sequence (which binds the transcription factor to DNA) is highlighted in yellow. In red is the single nucleotide substitution that occurred in the H-Cd NIL sequence.

Other domains involved in the bHLH transcriptional regulation are the basic domains which bind to DNA at a consensus hexanucleotide sequence known as the E-box [50]. Different families of bHLH proteins recognize different E-box consensus sequences: CAGCTG, CACCTG, CACGTG, CATGTTG, etc. *ORG2* (*bHLH38*) nucleotide sequences are also slightly different between the two near isogenic lines; a single nucleotide substitution (a T instead of a C) occurs in the E-box sequence of the H-Cd NIL (Figure 7). 

These sequence variations are translated into different proteins that probably have less efficiency to bond promoter regions and consequently in the regulation of *IREG2* and *NAS* genes. 

## 3. Materials and Methods

### 3.1. Plant Materials and Treatment

To identify the molecular mechanisms in response to Cd, we analyzed the transcriptomes of two near-isogenic lines (NILs) of durum wheat (*Triticum turgidum* L. subsp. *durum*) that differ in grain Cd accumulation: TL 8982-H (High-Cd NIL) and TL-8982 L (Low-Cd NIL) [28]. The breeding scheme is reported in Figure S1: “Near-Isogenic Line Breeding Scheme”.

For a rigorous Cd administration to the H-Cd and L-Cd NILs, a hydroponic system was set up.

The isogenic line seeds were externally sterilized and sprouted in cell culture dishes with humid filter paper, in the dark at 8 °C. After sprouting (about one week), seedlings were placed in cylindric pots (h = 50 cm, Ø = 10 cm) filled with perlite, soaked with deionized water and quickly moved to the hydroponic system as described by Harris and Taylor [16]. Three seedlings were planted in each pot and, for each treatment, three tubes were assembled (three biological replicates). The positions of each pot were completely random and regularly (every week) changed during cultivation The plants were irrigated with hydroponic solution at regular intervals (4 h) for 5 min in order to keep the perlite moistened, but to avoid stagnation. Plants were grown in two separate Fitotron^®^ Growth Rooms (Weiss Technik, Loughborough, UK) under controlled conditions [17].

The nutrient solution was prepared using reverse osmosis (RO) water (< 30 μS cm^−1^) and contained 1.1 mM KNO_3_, 3.0 mM [Ca(NO_3_)_2_ ·2H_2_O]NH_4_NO_3_, 0.2 mM NH_4_NO_3_, 1.2 mM K_2_HPO_4_ 0.04 g/l FeEDDHA, 2.0 mM MgSO_4_, 70 μM H_3_BO_3_, 1.2 μM Na_2_MoO_4_, 1.0 μM ZnSO_4_, 1.0 μM CuSO_4_, 10 μM MnSO_4_; the pH of the nutrient solution was kept constant between 5.5 and 6.0 and checked every 2 days. HEDTA was added to the nutrient solution to reproduce the environmental availability of free metals [51]. Hydroponic solution was continuously aerated. Treated plants were cultivated by adding 0.5 μM CdCl_2_ to the hydroponic solution. As reported by Harris and Taylor [16], such Cd treatment is not toxic for durum wheat roots. Roots and shoots were sampled in triplicate 50 days after sprouting, at the tillering stage. Roots were removed from the substrate and manually washed to remove the perlite from roots. Grains were collected at maturity. Samples were washed in RO water for 30 s. Root samples for mRNA sequencing were frozen in liquid nitrogen and then stored at −80 °C.

### 3.2. Biomass Analysis and Determination of Cd Concentration

Roots, shoots and grains were dried at 100 °C to a constant weight and, subsequently, dry weight was measured. The yield was quantified at maturity by an electronic laboratory balance (n = 3).

Cd concentration was quantified as described by Vergine and colleagues (2017). Samples of roots, shoots and grains were dried, and 0.1 g was processed in a solution with 6 mL of trace metal-grade concentrated HNO_3_ and 1 mL of 30% (*v*/*v*) H_2_O_2_, in a Milestone MLS 1200 MEGA microwave digestor (FKV, Sorisole, Italy). As described by Massadeh and Snook [52], deionized water (10 mL) was added after cooling, and the resulting solution was filtered using a Whatman filter paper 40 into a 25 mL volumetric flask. Cd was quantified by graphite furnace atomic absorption spectroscopy (GF-AAS, PinAAcle, PerkinElmer, Waltham, MA, USA). Quantitative analysis was completed by interpolating the calibration curves of metal standards. The method detection limit (MDL) was 0.08 μg L^−1^. Cd concentrations were obtained with the elimination of the average level observed in blank samples. Cd concentration was quantified if it was higher than the standard deviation σB of the blank; otherwise, a threshold value equal to σB was considered. If the concentration was below the MDL or not detectable above the average variability, a concentration value equal to the maximum between the MDL and σB was assumed.

### 3.3. RNA Isolation and mRNA Sequencing 

Total RNA was extracted from the root tissues using TRIZOL reagent [53]. To evaluate the quality and quantity of the extracted mRNA, several RNA dilutions were examined using the Agilent RNA 6000 nano Kit and Agilent Bioanalyzer 2100. RNA sequencing and bioinformatic analyses were performed by IGA Technology Services (Udine, Italy).

Three biological replicates of H-Cd and L-Cd NILs grown in hydroponic conditions without and with Cd (0.5 μM) were sequenced for each condition (24 samples). Sequencing was done in 100 bp single-end mode on a HiSeq2500 (Illumina, San Diego, CA, USA). Then, alignments were performed with TopHat2 [54,55] on a *Triticum* reference genome/transcriptome owned by IGA Technology Services, using default parameters. Fastq data have been deposited in the NCBI SRA archive under accession number SRR7938636. Median alignment rate was 85.7%. Homology-based functional annotation of *Triticum* genes was performed using BLAST on the *Arabidopsis thaliana* genome, after setting the E-value threshold at ≤10^−5^.

### 3.4. Identification of Differentially Expressed Genes

The relative abundances of mRNA copies were assessed by Cufflinks [56]. Pair-wise differential expression analysis was done by Cuffdiff [55]. Differentially expressed contigs were identified through a Welch *t*-test with Benjamini and Hochberg false discovery rate correction for multiple tests. A contig was flagged as differentially expressed (DEG) if it presented the following conditions: q-value (FDR-adjusted *p*-value) < 0.001, two-fold change (FC) in fragments per kilobase million (FPKM) value and an FPKM value of at least 5 in at least one samples. Applying a filtering process on FPKM > 5.0, we found that 37,952 genes were expressed in at least one condition.

qRT-PCR analysis was used to validate the RNA sequencing data. Eight arbitrary genes were selected for evaluation between RNA-seq data and qPCR. qRT-PCR reactions were run using SYBR Green fluorescence detection in a qPCR thermal cycler (ABI PRISM 7900HT, Applied Biosystems, Waltham, MA, USA). Each PCR tube was filled using 5 μL from a 0.2 ng/μL dilution of cDNA, 12.5 μL of SYBR Green PCR Master Mix (Applied Biosystems), 1 μM forward and reverse primers, in a total volume of 25 μL. The cycling conditions were: 10 min at 95 °C, followed by 40 cycles of 95 °C for 15 s and 60 °C for 1 min with the final dissociation at 95 °C for 15 s, 60 °C for 30 s and 95 °C for 15 s.

qRT-PCR needs internal references for gene expression data normalization. These genes are usually selected among the well-known housekeeping genes, such as actin, tubulin, etc. However, the expression of housekeeping genes is not always stable among genotypes, tissues or treatments. To find a gene(s) with a stable level of expression in the present work, the contigs showing a level of expression higher than 25 FKPM were considered. These contigs were unlisted according to their coefficient of variation (CV = standard deviation mean-1). The best three contigs were: contig21117 annotated as CCAAT-binding transcription factor C (NFYC-B6) (CV = 0.063), contig37744 coding for a lumazine synthase (CV 0.068) and contig24616 annotated as a cyanase (CV 0.077) (Table S1: “Expression data”). The stability of their expression across wheat samples was further checked by qRT-PCR and contig21117 (NFYC-B6) was selected as a reference gene due to its lowest CV in qRT-PCR, too.

RNA-seq data mining was performed using g:Profiler [33], a public web server for characterizing and manipulating gene lists resulting from high-throughput genomic data. g:Profiler allowed us to identify the statistical enrichment of functional categories among the lists of up- and downregulated genes, setting a *p*-value < 0.001. 

## 4. Conclusions

The evaluated hypotheses allow us to reveal some elements differentiating the L-Cd NILs which consequently could be involved in Cd compartmentalization at the root level. On the contrary, other genes, which are expressed only in H-Cd NILs, could have an important role in Cd translocation from root to shoot.

*Aquaporins pip1-2-like* and *aquaporin 1-5* genes are expressed only in H-Cd NILs and they should be involved in Cd translocation since aquaporin downregulation determines a reduction in water transport and, consequently, reduced Cd movement. The L-Cd NIL did not express these genes at all in CTRL or treated plants and, consequently, Cd translocation due to aquaporins should be reduced (or absent) in the L-Cd NIL. 

In the L-Cd NIL, we have identified several genes that are good candidates to explain greater Cd retention at the root level. One of them is the *HMT1* homologue gene, expressed only in the L-Cd genotype, which could confer resistance to Cd and could reduce Cd accumulation in grains, as reported in other studies. 

Genes sharing the same transcription factor binding sites are proven to be regulated by the same transcription factors. The Cd transporter homologues *IREG2* and *ZIFL1* were co-expressed with the genes of the mugineic acid pathway (*NASs*, *NAATs* and *DMASs*). Their upregulation in the L-Cd NIL was very strong (more than 100-fold), suggesting a central role in Cd response. These genes were upregulated in the H-Cd NIL, too, but the induction was lower (5–10-fold). These genes could act together in a complex way to finally reduce Cd translocation by chelation (mugineic acid pathway) and subsequent compartmentalization through Cd transporters (IREG2 and ZIFL1). The transcription factors bHLH29 and bHLH38 act together to regulate *NAS, NAAT* and *DMA* genes and the transporter IREG2. 

The contigs corresponding to *bHLH29* and *bHLH38* were similarly upregulated in L-Cd and H-Cd NILs, but the mutation on the hexanucleotide motif in the H-Cd NIL *bHLH38* gene could cause less binding affinity to *NAS*, *NAAT*, *DMA*, *IREG2* and *ZIFL1* genes, resulting in reduced immobilization of Cd at the root level and, consequently, higher accumulation in grains.

In conclusion, we have identified several genes putatively involved in or responsible for the molecular mechanisms for Cd intake and translocation which in turn should determine Cd accumulation in durum wheat kernels. In any case, only further work, in particular with a yeast two-hybrid system or with a knockout mutant of Arabidopsis, will prove which genes are involved in Cd response and accumulation in plant tissues. The target genes of these new experiments should be the transcription factors *bHLH38* and *bHLH29*, *NAS*, *NAAT* and *DMAS* genes and the transporters *IREG2* and *ZIFL1*. 

These studies will help to find new low Cd accumulating genotypes useful for durum wheat cultivation in Cd-contaminated soils or that can be used as starting material for breeding programs.

## Figures and Tables

**Table 1:** List of genes constitutively expressed in the L-Cd NIL, but not expressed in the H-Cd NIL. In the first column is the contig name and in the following columns are reported the expression levels (FPKM). In the last column, the gene annotation based on BLAST sequence analysis is reported.

Contig ID	L-Cd CTRL	L-Cd +Cd	H-Cd CTRL	H-Cd +Cd	Annotation
*contig19712*	27.4	31.2	2.4	2.1	Cyclophilin (*AT3G66654*)
*contig23984*	76.5	49.8	0.1	0.1	no hits—unknown
*contig5761*	36.4	25.7	4.9	4.0	3-mercaptopyruvate sulfurtransferase (*AT1G79230*)
*contig90107*	57.1	30.7	0.0	0.0	no hits—unknown
*contig9490*	53.2	43.9	2.8	0.2	ABC transporter 3-like (*AT1G64550*)

**Table 2:** List of genes constitutively expressed in the H-Cd NIL, but not expressed in the L-Cd NIL. In the first column is the contig name and in the following columns are reported the expression levels (FPKM). In the last column, the gene annotation based on BLAST sequence analysis is reported.

Contig ID	L-Cd CTRL	L-Cd +Cd	H-Cd CTRL	H-Cd +Cd	Annotation
*contig13191*	0.1	0.3	97.8	34.2	aquaporin pip1-2 (*AT1G01620*)
*contig17660*	0.3	0.2	148.0	65.1	aquaporin pip1-5 (*AT4G00430*)
*contig17681*	0.0	0.1	33.2	27.0	no hits—unknown
*contig20402*	0.1	0.0	62.1	58.1	no hits—unknown
*contig35423*	0.0	0.1	70.6	43.7	Ribosomal protein (*AT1G33140*)
*contig3675*	0.4	0.4	104.1	84.4	no hits—unknown
*contig48835*	4.4	4.4	62.9	103.4	no hits—unknown
*contig5342*	3.3	3.9	52.2	67.4	RNA ligase (*AT4G18930*)
*contig1280*	3.1	1.9	30.9	32.6	transcription elongation factor (*AT1G32130*)

**Table 3:** List of genes more upregulated in roots of L-Cd NIL and annotated as *nicotianamine synthase* (*NAS*) genes. In the first column is the contig name and in the following columns are the expression levels (FPKM). The FC columns report fold changes. In the last column is the gene annotation based on BLAST sequence analysis.

	L-Cd			H-Cd				
Contig ID	CTRL	0.5 µM	FC	CTRL	0.5 µM	FC	AGI Code	Annotation
*contig26634*	7.8	604.5	77.1	24.3	240.7	9.9		NAS 1
*contig55580*	3.6	707.5	195.8	20.5	383.4	18.7		NAS 1
*contig44671*	5.4	774.2	143.6	49.4	437.2	8.8		NAS 2
*contig18293*	1.8	502.3	278.2	11.1	149.3	13.5	*AT5G56080*	NAS 2
*contig18375*	23.5	653.3	27.8	64.6	386.0	6.0	*AT5G56080*	NAS 2
*contig18424*	10.6	1178.3	111.5	41.5	522.9	12.6	*AT1G09240*	NAS 3
*contig33892*	1.1	623.2	572.5	10.0	262.0	26.3	*AT1G09240*	NAS 3
*contig40805*	9.8	629.4	64.2	55.9	348.5	6.2	*AT1G09240*	NAS 3
*contig5670*	1.1	179.2	158.9	5.2	23.2	4.5	*AT1G09240*	NAS 3
*contig6866*	7.6	663.0	87.8	51.9	281.9	5.4	*AT1G09240*	NAS 3
*contig16879*	0.8	155.6	204.0	5.2	68.2	13.0		NAS 4
*contig36649*	7.8	482.7	61.8	19.6	183.5	9.4		NAS 4
*contig13016*	2.1	391.5	186.2	30.2	114.6	3.8	*AT1G56430*	NAS 4
*contig18348*	2.4	626.8	264.5	25.2	257.9	10.2	*AT1G56430*	NAS 4
*contig27606*	7.4	321.2	43.7	31.3	106.5	3.4	*AT1G56430*	NAS 4
*contig46013*	7.5	1488.7	197.4	64.4	306.9	4.8	*AT1G56430*	NAS 4
*contig51008*	1.2	369.2	318.0	19.5	85.3	4.4	*AT1G56430*	NAS 4
*contig5669*	12.5	845.6	67.6	44.2	248.1	5.6	*AT1G56430*	NAS 4
*contig56695*	1.7	169.2	102.4	12.5	75.1	6.0	*AT1G56430*	NAS 4
*contig62096*	6.4	778.0	122.2	39.4	270.5	6.9	*AT1G56430*	NAS 4
*contig6432*	5.3	1071.6	200.7	81.6	218.6	2.7	*AT1G56430*	NAS 4
*contig35044*	10.3	691.1	67.3	49.2	419.8	8.5		NAS 5B
	CTRL	0.5 µM	FC	CTRL	0.5 µM	FC		
Mean	6.3	632.1	161.5	34.4	245.0	8.7		

**Table 4:** Expression level of the contigs annotated as *NAAT* and *DMAS*. Expression level of the genes coding for the enzymes NAAT and DMAS involved in mugineic acid biosynthesis. In the first column is the contig name and in the following columns are the expression levels (FPKM). In the last column, the gene annotation based on BLAST sequence analysis is reported.

	L-Cd			H-Cd				
Contig ID	CTRL	0.5 µM	FC	CTRL	0.5 µM	FC	AGI Code	Annotation
*contig24852*	0.7	246.9	356.6	23.8	103.0	4.3	*AT2G20610*	NAAT
*contig7573*	1.0	209.9	209.6	10.9	117.3	10.7	*AT5G53970*	NAAT
*contig20039*	3.2	462.9	145.4	49.6	287.9	5.8	*AT5G53970*	NAAT
*contig40067*	21.9	274.7	12.6	33.9	175.8	5.2	*AT1G59960*	DMAS
*contig10233*	11.4	253.8	22.3	22.9	139.9	6.1	*AT1G59960*	DMAS
	CTRL	0.5 µM	FC	CTRL	0.5 µM	FC		
Mean	7.6	289.6	149.3	28.2	164.8	6.4		

## Data Availability

Data have been deposited in the NCBI SRA archive under accession number SRR7938636.

## Supplementary Materials

The following are available online at https://www.mdpi.com/article/10.3390/ijms22147343/s1.
