# Supplementary figures and images for: Molecular Responses to Cadmium Exposure in Two Contrasting Durum Wheat Genotypes
Source: Int J Mol Sci. 2021 Jul 8;22(14):7343. doi: 10.3390/ijms22147343 (PMC8306872; doi:10.3390/ijms22147343)

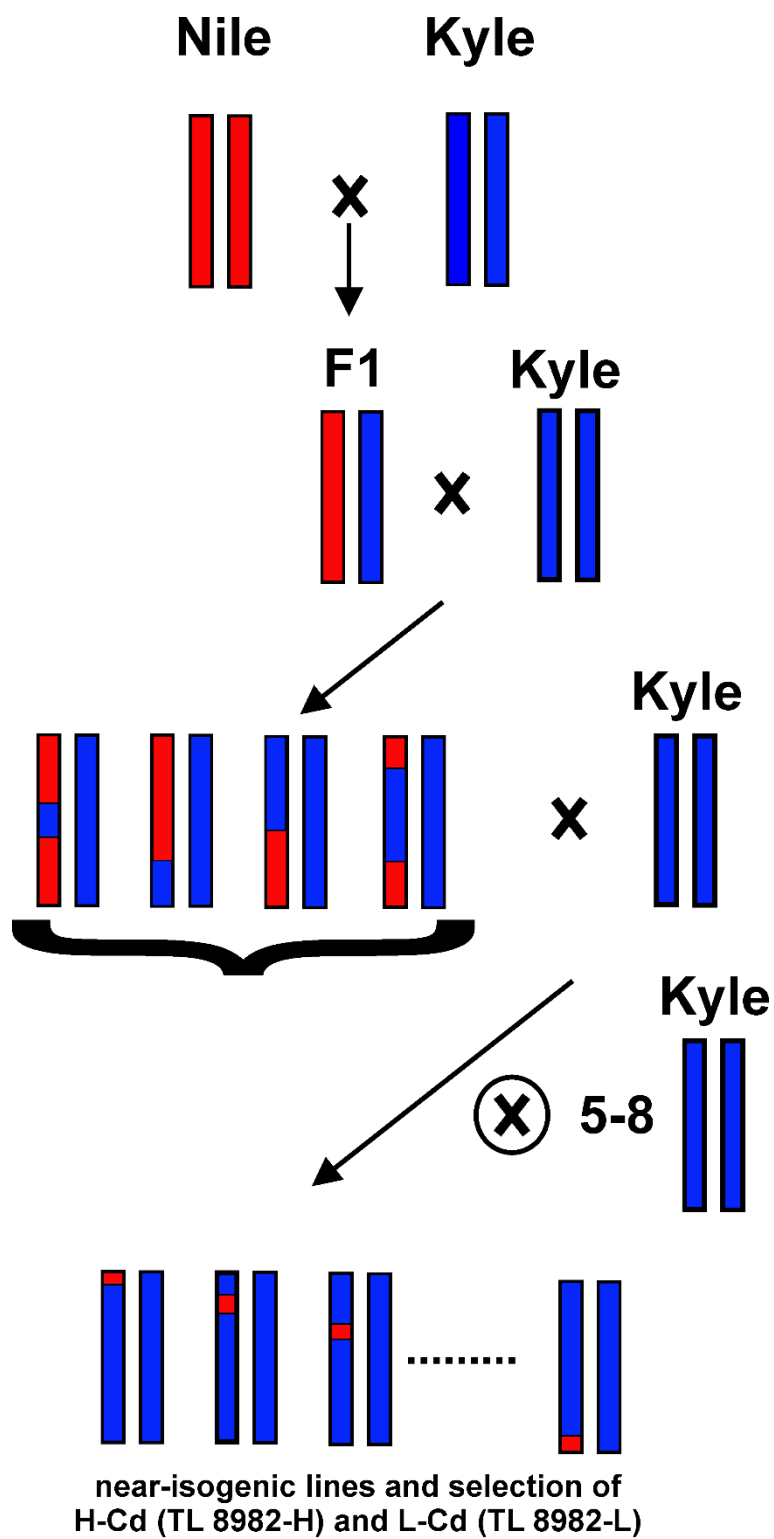

Figure S1. Near-isogenic line breeding scheme.

Supplement: Supplementary file 1 [file ijms-22-07343-s001.zip › Figure S1_Near-Isogenic Line Breeding Scheme.pdf]
